# Robustness of the Ferret Model for Influenza Risk Assessment Studies: a Cross-Laboratory Exercise

**DOI:** 10.1128/mbio.01174-22

**Published:** 2022-07-11

**Authors:** Jessica A. Belser, Eric H. Y. Lau, Wendy Barclay, Ian G. Barr, Hualan Chen, Ron A. M. Fouchier, Masato Hatta, Sander Herfst, Yoshihiro Kawaoka, Seema S. Lakdawala, Leo Yi Yang Lee, Gabriele Neumann, Malik Peiris, Daniel R. Perez, Charles Russell, Kanta Subbarao, Troy C. Sutton, Richard J. Webby, Huanliang Yang, Hui-Ling Yen, Working group on the standardization of the ferret model for influenza risk assessment

**Affiliations:** a Influenza Division, Centers for Disease Control and Prevention, Atlanta, Georgia, USA; b School of Public Health, Li Ka Shing Faculty of Medicine, The University of Hong Kong, Hong Kong SAR, China; c Imperial College, London, United Kingdom; d WHO Collaborating Centre for Reference and Research on Influenza, VIDRL, Doherty Institute, Melbourne, Australia; e State Key Laboratory of Veterinary Biotechnology, Harbin Veterinary Research Institute, Chinese Academy of Agricultural Sciences, Harbin, China; f Department of Viroscience, Erasmus MC, Rotterdam, The Netherlands; g University of Wisconsin, Madison, Wisconsin, USA; h University of Pittsburgh, Pittsburgh, Pennsylvania, USA; i University of Georgia, Athens, Georgia, USA; j Department of Infectious Diseases, St. Jude Children’s Research Hospital, Memphis, Tennessee, USA; k Department of Microbiology and Immunology, University of Melbourne, Doherty Institute, Melbourne, Australia; l Pennsylvania State University, University Park, Pennsylvania, USA; Icahn School of Medicine at Mount Sinai; Centers for Disease Control and Prevention, USA; School of Public Health, The University of Hong Kong, China; Imperial College, UK; WHO Collaborating Centre for Reference and Research on Influenza, Doherty Institute, Australia; Harbin Veterinary Research Institute, Chinese Academy of Agricultural Sciences, China; Erasmus MC, The Netherlands; University of Wisconsin, Madison, WI; University of Pittsburgh, Pittsburgh, PA; University of Georgia, USA; St. Jude Children’s Research Hospital, USA; Pennsylvania State University, USA

**Keywords:** assay standardization, ferret model, influenza, pandemic risk assessment, transmissibility

## Abstract

Past pandemic influenza viruses with sustained human-to-human transmissibility have emerged from animal influenza viruses. Employment of experimental models to assess the pandemic risk of emerging zoonotic influenza viruses provides critical information supporting public health efforts. Ferret transmission experiments have been utilized to predict the human-to-human transmission potential of novel influenza viruses. However, small sample sizes and a lack of standardized protocols can introduce interlaboratory variability, complicating interpretation of transmission experimental data. To assess the range of variation in ferret transmission experiments, a global exercise was conducted by 11 laboratories using two common stock H1N1 influenza viruses with different transmission characteristics in ferrets. Parameters known to affect transmission were standardized, including the inoculation route, dose, and volume, as well as a strict 1:1 donor/contact ratio for respiratory droplet transmission. Additional host and environmental parameters likely to affect influenza transmission kinetics were monitored and analyzed. The overall transmission outcomes for both viruses across 11 laboratories were concordant, suggesting the robustness of the ferret model for zoonotic influenza risk assessment. Among environmental parameters that varied across laboratories, donor-to-contact airflow directionality was associated with increased transmissibility. To attain high confidence in identifying viruses with moderate to high transmissibility or low transmissibility under a smaller number of participating laboratories, our analyses support the notion that as few as three but as many as five laboratories, respectively, would need to independently perform viral transmission experiments with concordant results. This exercise facilitates the development of a more homogenous protocol for ferret transmission experiments that are employed for the purposes of risk assessment.

## INTRODUCTION

Pandemic influenza viruses with novel antigenicity and sustained transmissibility in humans arise periodically from animal origin influenza viruses and may result in profound public health and social-economic impact. Animal influenza viruses that have repeatedly caused zoonotic inflections at the human-animal interface may have increased pandemic risk. To ascertain their pandemic risk, the WHO and CDC have developed risk assessment tools to evaluate the human-to-human transmissibility of influenza virus (likelihood of pandemic emergence) and the capacity of the virus to cause severe disease in humans (potential public health impact) (1, 2). These characterization efforts are undertaken by laboratories worldwide, employing zoonotic virus strains and experimental protocols that are similar but not uniform, which may lead to variation in the experimental results. To date, limited assessments of interlaboratory variability have been conducted for influenza virus serological assays (3–6), but not for other *in vitro* or *in vivo* assays.

One of the essential components in pandemic risk assessment is to evaluate the human-to-human transmission potential of zoonotic influenza viruses in suitable animal models. Ferrets have been used as a surrogate model for studying the transmission mechanisms of influenza viruses (7–10), as they are naturally susceptible to infection with human and zoonotic influenza viruses, exhibit clinical signs during infection which closely resemble those of humans, and support influenza virus transmission via modes similar to those in humans. In particular, the respiratory droplet transmissibility of a specific influenza strain among ferrets often correlates with its transmission potential in humans (11). Therefore, ferrets are commonly used for assessing the pandemic potential of newly emerged zoonotic influenza viruses, and data from these experiments inform formal risk assessment rubrics (1, 2).

The transmission potential of influenza viruses is determined by multiple viral, host, and environmental parameters. As the ferret model becomes commonly employed in laboratories worldwide, there is an underappreciated heterogeneity among established experimental protocols and facility setups across different laboratories, which may lead to variable results between transmission experiments performed (12). Some of these variables, such as the dose, volume, and route of inoculation and animal age, have been confirmed to affect the kinetics of virus infection, replication, and transmission in the ferret model (13–15). However, the impact of other parameters, such as virus propagation procedures, caging designs, airflow directionality and number of air exchanges, and environmental conditions such as relative humidity, is largely unknown. Consequently, interpretation of results from ferret transmission experiments can represent a challenge when comparing data generated from multiple laboratories, even when the same virus strain or subtype is being investigated (16). Due to the statistical limitations on small sample sizes in ferret experiments, variations in experimental protocols, and the high potential for strain-specific variability, discrepancies between ferret transmission results across laboratories have been reported for the A(H1N1)pdm09 pandemic influenza virus (17–23) and the Asian A(H7N9) avian influenza virus, which has caused zoonotic infections in humans since 2013 (24–29). Agencies in charge of pandemic risk assessment will often assess the pandemic potential of emerging virus subtypes as an aggregate of multiple viruses tested (30–32). As many public health efforts require cross-laboratory risk assessment studies for newly emerged zoonotic influenza viruses (33) and antiviral efficacy studies aiming to block influenza transmission between ferrets (34), a greater understanding of variability in transmission results obtained between independent groups is critical.

To assess the variability of ferret transmission results across laboratories under established protocols, we performed a global exercise using two common stock influenza viruses that possess different transmission characteristics in ferrets. Eleven independent laboratories inoculated ferrets with these stock viruses under uniform conditions; parameters known to affect influenza transmission kinetics were controlled in the experimental protocols while other potential parameters were carefully monitored and recorded, both prior to and during the transmission experiments. All aggregated data from these experiments were de-identified and analyzed by an independent statistician. To inform future risk assessment activities, the confidence of drawing conclusions on virus transmissibility with concordant or discordant outcomes from multiple laboratories was also investigated. By assessing the range of variation present among ferret transmission experiments performed under established experimental protocols, this global exercise provides helpful guidance for data interpretation when cross-laboratory results are to be compared. The relatively concordant transmission results across 11 laboratories suggest that the ferret model is highly robust for influenza pandemic risk assessment studies under the semistandardized conditions employed in this study. Furthermore, analyses investigating the role of host and environmental parameters as they contribute to virus transmission kinetics and outcomes are valuable for both current risk assessment activities and evaluation of countermeasures to block influenza transmission.

## RESULTS

### Transmissibility of human A(H1N1)pdm09 virus.

**FIG 1:**
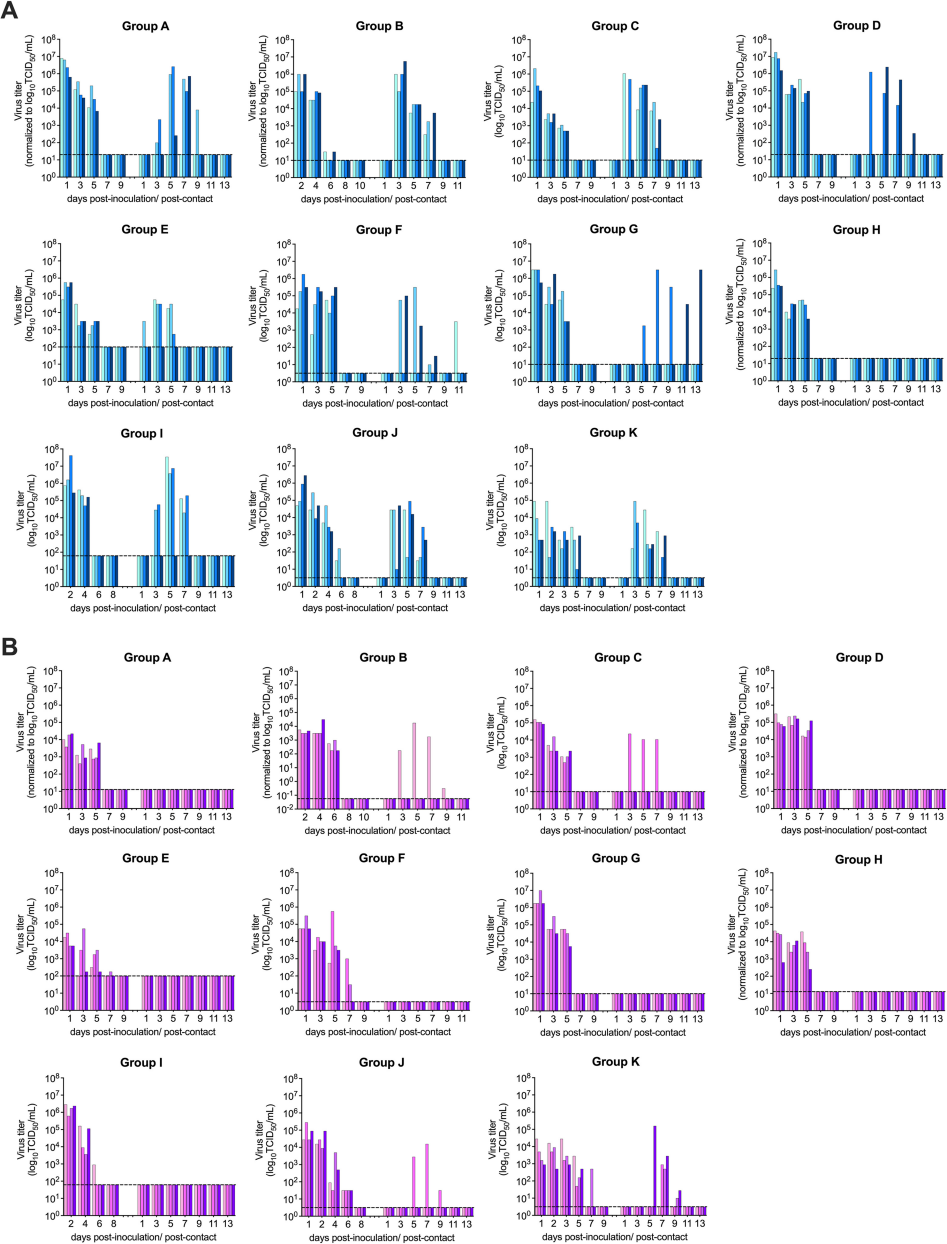
Transmission kinetics of A(H1N1) viruses in ferrets. (A) Normalized viral loads of donors (left bars) and aerosol contact ferrets (right bars) after inoculation or exposure to A(H1N1)pdm09 virus Cal/09. (B) Normalized viral loads of donors (left bars) and aerosol contact ferrets (right bars) after inoculation or exposure to avian H1N1 virus ruddy turnstone/09. Nasal washes (all groups except group F) or throat swabs (group F) were sampled to determine infectious viral loads, which were normalized to log_10_ TCID_50_ per milliliter. Each bar represents an individual ferret. The limit of detection is indicated with a dashed line.

To evaluate potential heterogeneity in the transmission results between 11 laboratories, we first compared the transmissibility of a cell-grown isolate of the 2009 pandemic A(H1N1)pdm09 virus A/California/7/2009 (Cal/09), representative of early 2009 pandemic isolates and anticipated to exhibit moderate to high respiratory droplet transmissibility (17, 21, 22, 35). Transmissibility was evaluated with 4 donor-contact pairs at a 1:1 ratio in each laboratory. Transmission to exposed respiratory droplet contact ferrets was defined by detection of infectious virus or seroconversion to the homologous virus in postexposure sera. Following establishment of contact with donor ferrets 24 h postinoculation, detection of infectious virus and seroconversion in contacts was observed in 10/11 and 11/11 laboratories, respectively, with the reported transmission frequency ranging from 50 to 100% (Table 1). One out of 11 laboratories determined viral loads in nasal swabs and throat swabs (group F, with throat swab viral loads used for subsequent analysis), while the other laboratories determined viral loads in nasal washes. Employing both virological and serological results, by Fisher’s exact test of homogeneity, there was no significant difference in the transmission outcomes across labs with this virus (*P* = 0.797). Collectively, infectious virus was detected from the nasal wash or throat swabs of 72.7% (32/44) of exposed contacts, and seroconversion of contact ferrets to homologous virus was detected from 79.5% (35/44) of exposed contacts. To allow comparison of the effect of viral load on transmissibility, viral titer units from nasal wash/throat swab samples (inclusive of 50% tissue culture infectious dose [TCID_50_], PFU, and 50% egg infectious dose [EID_50_] units [see Fig. S1 in the supplemental material]) were normalized to TCID_50_ units (Fig. 1), employing strain-specific conversions prior to analyses (Table S1). From the inoculated donor ferrets, the peak viral titers detected in the nasal washes or throat swabs were at 5.72 ± 0.95 log_10_ TCID_50_/mL (mean ± standard deviation [SD]) after normalization, with the peak titers detected from 95.5% (42/44) of donors at 1 or 2 days postinoculation (dpi) (first sampling time point) followed by a decline of infectious titer over time (Fig. 1A). Area under the curve (AUC) after normalization was calculated to approximate total viral load shed by the Cal/09-inoculated donors, and the log_10_ AUC was found to be 5.84 ± 0.89 (mean ± SD).

**TABLE 1:** Summary of virus transmissibility results from all laboratories^*a*^

Group	A(H1N1)pdm09 virus A/California/7/2009			A(H1N1) avian influenza virus A/ruddy turnstone/Delaware/300/20/2009		
	Viral load of inoculated donors (AUC)	Transmission to aerosol contacts (no./total)		Viral load of inoculated donors (AUC)	Transmission to aerosol contacts (no./total)	
		Virus detection	Seroconversion		Virus detection	Seroconversion
A	6.51 ± 0.49	3/4	3/4	4.28 ± 0.35	0/4	0/4
B	5.70 ± 0.42	4/4	4/4	4.25 ± 0.39	1/4	1/4
C	5.30 ± 0.78	4/4	4/4	5.10 ± 0.11	1/4	1/4
D	6.86 ± 0.40	2/4	2/4	5.73 ± 0.21	0/4	0/4
E	5.53 ± 0.32	3/4	3/4	4.43 ± 0.56	0/4	0/4
F	5.77 ± 0.60	3/4	3/4	5.34 ± 0.60	0/4	0/3
G	6.57 ± 0.06	2/4	2/4	6.48 ± 0.37	0/4	0/4
H	5.82 ± 0.43	0/4	3/4	4.72 ± 0.31	0/4	0/4
I	6.48 ± 0.80	3/4	3/4	6.24 ± 0.31	0/4	0/4
J	5.62 ± 0.54	4/4	4/4	4.92 ± 0.39	1/4	1/4
K	4.07 ± 0.72	4/4	4/4	4.15 ± 0.57	3/4	3/4

a Viral loads detected from inoculated donors were normalized to log_10_ TCID_50_ per milliliter across laboratories; area under the curve (AUC) was determined to approximate total viral load. Transmission to aerosol contacts was evaluated using detection of infectious viruses in respiratory specimens and by seroconversion at the end of the study using hemagglutination inhibition assay.

10.1128/mbio.01174-22.2 TABLE S1 Summary of titration methodology by each laboratory. Download Table S1, DOCX file, 0.03 MB. Copyright © 2022 Belser et al. 2022 Belser et al. https://creativecommons.org/licenses/by/4.0/ This content is distributed under the terms of the Creative Commons Attribution 4.0 International license.

10.1128/mbio.01174-22.9 FIG S1 Transmission kinetics of H1N1 viruses. Nasal washes (all groups except group F) or throat swabs (group F) were sampled from donor (left bars) and aerosol contact (right bars) ferrets to determine infectious viral loads following inoculation with A/California/7/2009 (A) or A/ruddy turnstone/DE/300/2009 (B); titers are reported as log_10_ PFU per milliliter (groups A, D, and H), log_10_ TCID_50_ per milliliter (groups C, E, F, G, I, J, and K), or log_10_ EID_50_ per milliliter (group B). The limit of detection for each graph is reported in Table S1. Download FIG S1, DOCX file, 0.4 MB. Copyright © 2022 Belser et al. 2022 Belser et al. https://creativecommons.org/licenses/by/4.0/ This content is distributed under the terms of the Creative Commons Attribution 4.0 International license.

**FIG 2:**
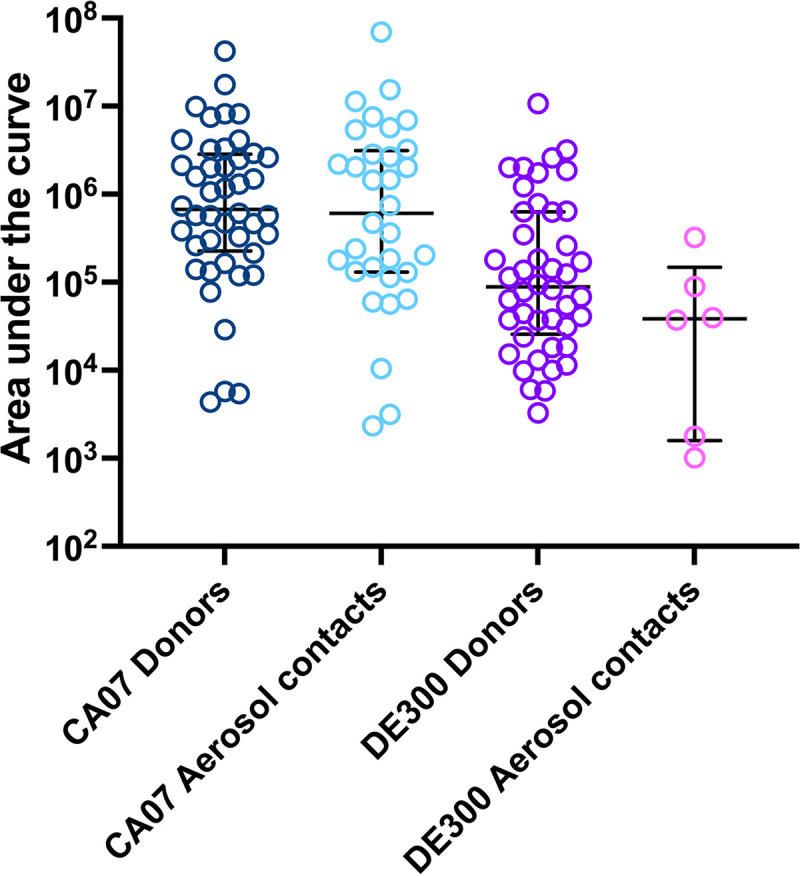
Area under the curve of infectious viral loads detected from inoculated donors or infected contacts. Data points represent AUC values from individual ferrets from which infectious virus was detected. **, *P* < 0.01; ****, *P* < 0.0001 (Mann-Whitney test).

Next, to evaluate the transmission efficiency, the serial interval (first detection of viral shedding in contacts postexposure from specimens collected every other day) was calculated for each infected contact ferret. The serial interval was 1 day for 3.1% (1/32) of the Cal/09-infected contact ferrets, followed by 3 days for 68.8% (22/32), 5 days for 21.9% (7/32), and 11 days for 6.3% (2/32), with a median serial interval of 3 days postcontact. Peak viral titers detected in the contact nasal washes or throat swabs were at 5.41 ± 1.06 log_10_ TCID_50_s/mL (mean ± SD) after normalization, with peak titers detected from 50% (16/32) and 34.4% (11/32) of infected contacts at 3 dpi and 5 dpi, respectively. Altogether, the AUC for Cal/09-infected contact ferrets was 5.75 ± 1.05, comparable to that of the Cal/09 virus-inoculated donors (Mann-Whitney test, *P* = 0.6547) (Fig. 2).

### Transmissibility of avian A(H1N1) influenza virus.

We further evaluated the range of heterogeneity present in transmission results when using the A/ruddy turnstone/Delaware/300/20/09 (ruddy turnstone/09) A(H1N1) avian influenza virus (36, 37), which has been reported to transmit in ferrets via respiratory droplets under the experimental setting of donor/direct contact/respiratory droplet contact at a 1:1:1 ratio but not at a 1:1 donor/respiratory droplet contact ratio (R. Fouchier, unpublished data) (36, 37). Here, the experimental setup and conditions were identical to those for assessing Cal/09 virus transmissibility, including a donor/respiratory droplet contact 1:1 ratio with no direct contact ferret. Transmission of an egg-derived isolate of ruddy turnstone/09 virus to exposed respiratory droplet contacts was observed in only 4 out of the 11 laboratories, with the transmission frequencies ranging from 25 to 75% across these four laboratories (Table 1). There were greater differences in the ruddy turnstone/09 virus transmission outcomes across 11 laboratories than for Cal/09 virus, but the difference did not reach statistical significance by Fisher’s exact test of homogeneity (*P* = 0.068). Viral shedding and seroconversion to ruddy turnstone/09 virus were detected from 6/43 exposed contact ferrets across all laboratories, resulting in a transmission efficiency of 14.0%, which was significantly lower than that of Cal/09 virus (72.3%; paired *t* test, *P* < 0.001).

From the inoculated donor ferrets, the peak viral titers detected in the nasal washes or throat swabs were at 4.85 ± 0.94 log_10_ TCID_50_/mL (mean ± SD) after normalization, which was significantly lower than those detected in the Cal/09-inoculated donors (Mann-Whitney test, *P* < 0.0001). Peak titers were detected from 88.6% (39/44) donors at the first sampling time point (1 or 2 dpi), followed by a decline of infectious titer over time (Fig. 1B). The log_10_ AUC of ruddy turnstone/09 virus-inoculated ferrets was 5.06 ± 1.86 (mean ± SD), significantly lower than for those inoculated with the Cal/09 virus (Mann-Whitney test, *P* < 0.0001) (Fig. 2). Overall, ruddy turnstone/09 virus-inoculated donor ferrets shed lower titers of infectious virus than the Cal/09 virus-inoculated donors.

In contrast to the transmission efficiency of Cal/09 virus, with a median serial interval of 3 days, for the ruddy turnstone/09 transmission experiments, the serial intervals were 3 days, 5 days, and 7 days for 33.3% (2/6), 33.3% (2/6), and 33.3% (2/6) of the infected contact ferrets, respectively, with the median serial interval at 5 days. Peak viral loads (3.94 ± 0.94 log_10_ TCID_50_/mL [mean ± SD]) detected from the six infected contact ferrets were lower than for the Cal/09-infected contact ferrets (Mann-Whitney test, *P* = 0.0022). Peak titers were detected from 16.7% (1/6), 33.3% (2/6), and 50% (3/6) of infected contacts at 3 dpi, 5 dpi, and 7 dpi, respectively. Furthermore, ruddy turnstone/09 virus-infected contact ferrets shed significantly less infectious virus (log_10_ AUC, 4.31 ± 0.98 [mean ± SD]) than did those animals directly inoculated with Cal/09 virus (Mann-Whitney test, *P* = 0.0033) (Fig. 2). Taken together, the results show that there was a longer serial interval and lower infectious virus shed by ruddy turnstone/09 virus-exposed contact ferrets than for those exposed to Cal/09 virus.

### Factors associated with ruddy turnstone/09 virus transmissibility.

By standardizing the source stock virus, dose and volume of inoculation, and donor-to-contact ratio, we show that while infrequent discordant results were documented, the transmission outcomes of Cal/09 and ruddy turnstone/09 viruses independently performed by 11 laboratories were in general concordant, despite variabilities in the laboratory settings that were not standardized in the experiments (Tables S2, S3, and S4). As the transmission outcomes for the highly transmissible Cal/09 virus were more concordant than for the less transmissible ruddy turnstone/09 virus, we attempted to examine if any variable, including those not standardized between laboratories, may have been associated with differences in ruddy turnstone/09 virus transmissibility results.

10.1128/mbio.01174-22.3 TABLE S2 Ferret source and health status prior to study. Download Table S2, DOCX file, 0.03 MB. Copyright © 2022 Belser et al. 2022 Belser et al. https://creativecommons.org/licenses/by/4.0/ This content is distributed under the terms of the Creative Commons Attribution 4.0 International license.

10.1128/mbio.01174-22.4 TABLE S3 Caging and physical environment. Download Table S3, DOCX file, 0.03 MB. Copyright © 2022 Belser et al. 2022 Belser et al. https://creativecommons.org/licenses/by/4.0/ This content is distributed under the terms of the Creative Commons Attribution 4.0 International license.

10.1128/mbio.01174-22.5 TABLE S4 Environmental summaries for transmission experiments. Download Table S4, DOCX file, 0.03 MB. Copyright © 2022 Belser et al. 2022 Belser et al. https://creativecommons.org/licenses/by/4.0/ This content is distributed under the terms of the Creative Commons Attribution 4.0 International license.

Univariable logistic regression was performed to first evaluate if donor viral shedding kinetics were linked to ruddy turnstone/09 virus transmission efficiency. However, several parameters, including AUC (*P* = 0.193), peak viral titer (*P* = 0.197), and days to peak titer (*P* = 0.473), were not statistically associated with different transmission outcomes observed between laboratories (Table 2), indicating that differences observed between laboratories were not attributable to virological measurements.

**TABLE 2:** Parameters associated with transmission of ruddy turnstone/09^*a*^

Parameter	Unadjusted OR (95% CI)	*P* value
Donor viral load (log_10_ AUC)	0.18 (0.01, 2.37)	0.193
Donor peak titer (log_10_TCID_50_/mL)	0.56 (0.23, 1.35)	0.197
Donor time to peak titer (dpi)	0.28 (0.01, 8.88)	0.473
Air change (per 10 ACH)	0.61 (0.19, 1.96)	0.408
Directional airflow to contacts (reference: without directional airflow)	4.00 (0.27, 60.32)	0.317
Temp (per 0.1°C)	1.01 (0.85, 1.21)	0.874
Relative humidity^*b*^ (%)	0.94 (0.83, 1.06)	0.318
Absolute humidity^*b*^ (per g/m^3^)	0.71 (0.36, 1.42)	0.337
Distance between cages (cm)	1.01 (0.63, 1.60)	0.983

a Laboratories which detected transmission of ruddy turnstone/09 (i.e., infection of more than one ferret out of four contacts) were compared to laboratories which detected no transmission of the virus. Parameters that may affect transmission kinetics in ferrets were analyzed using univariable logistic regression. All parameters except directional airflow to contacts were numeric.

b No U-shape association with mean relative humidity or mean absolute humidity was observed by testing a quadratic term in the logistic regression model (*P* values > 0.15).

Numerous studies have indicated a role for environmental parameters in virus transmissibility (38, 39). Room temperature was generally consistent across all groups, with means of daily recordings within 3°C for all experiments performed (20.5 to 23.2°C) (Table S4). In contrast, the relative humidity (RH) reported between groups varied widely. Mean recordings over the entirety of each experiment ranged from 32.7 to 77% between groups, with daily observations spanning 30 to 100% RH. Furthermore, there was high variability in day-to-day readings over the 14-day experimental period, with low and high daily readings over a 14-day period varying 1 to 60% between different groups. Despite this variability, there was no statistically significant association between transmission of ruddy turnstone/09 virus and temperature, relative humidity, or absolute humidity (Table 2).

Experimental cage setups varied widely between different groups, with extensive heterogeneity present with regard to cage dimensions, airflow directionality and air changes per hour (ACH), distance between cages, and other parameters (Table S3). Groups employing caging with airflow directionality from inoculated to contact cages more frequently reported moderate to high transmissibility of both viruses, defined as a *P* of ≥50% transmission events per total pairs of ferret (e.g., transmission confirmed in ≥2 out of 4 pairs of contact ferrets) compared with groups lacking this airflow directionality (6/6 versus 3/5 groups for Cal/09 virus and 3/6 versus 1/5 groups for ruddy turnstone/09 virus); however, these findings did not reach statistical significance (*P* > 0.3 for both) (Table 2). Other specific features of cage setups, including distance between inoculated and contact cages and ACH, were also not statistically linked to the ruddy turnstone/09 transmission outcomes (*P* > 0.4 for both) (Table 2). Taken together, the findings show that despite substantial heterogeneity in numerous nonstandardized parameters in experimental setups employed between groups, no one feature was identified as modulating transmission outcomes to a significant degree.

### Factors associated with viral pathogenicity.

All ferrets inoculated with either Cal/09 or ruddy turnstone/09 were productively infected; however, measurements of morbidity varied between groups for both viruses. Among Cal/09 virus-inoculated ferrets, mean maximum weight loss and peak rise in body temperature between groups ranged from <1.0 to 15.6% and 0.6 to 2.1°C, respectively (Table S5 and Fig. S2A). Following ruddy turnstone/09 virus inoculation, infected ferrets generally exhibited greater mean maximum weight loss (up to 19.6%) and transient fevers (up to 3°C) (Table S6 and Fig. S2B) than did ferrets with Cal/09 virus infections; ruddy turnstone/09-inoculated ferrets reached humane experimental endpoints in 2/11 groups. The coefficients of variation between mean maximum weight loss reported between groups were generally similar (56% and 52% for Cal/09 and ruddy turnstone/09 viruses, respectively). No commonality with increased morbidity and ferret vendor, gender, or preinoculation body weight was identified. Furthermore, no association was found between morbidity and viral load (peak titer or AUC) or other environmental parameters, with the exception of room temperature (with higher mean room temperatures associated with greater mean weight loss) (Table S7).

10.1128/mbio.01174-22.6 TABLE S5 Clinical signs of donor ferrets following inoculation with Cal/09 virus. Download Table S5, DOCX file, 0.03 MB. Copyright © 2022 Belser et al. 2022 Belser et al. https://creativecommons.org/licenses/by/4.0/ This content is distributed under the terms of the Creative Commons Attribution 4.0 International license.

10.1128/mbio.01174-22.7 TABLE S6 Clinical signs of donor ferrets following inoculation with ruddy turnstone/09 virus. Download Table S6, DOCX file, 0.03 MB. Copyright © 2022 Belser et al. 2022 Belser et al. https://creativecommons.org/licenses/by/4.0/ This content is distributed under the terms of the Creative Commons Attribution 4.0 International license.

10.1128/mbio.01174-22.8 TABLE S7 Parameters associated with percent weight changes of Cal/09- and ruddy turnstone/09-inoculated donors by univariable linear regression. Download Table S7, DOCX file, 0.03 MB. Copyright © 2022 Belser et al. 2022 Belser et al. https://creativecommons.org/licenses/by/4.0/ This content is distributed under the terms of the Creative Commons Attribution 4.0 International license.

10.1128/mbio.01174-22.10 FIG S2 Weight changes of donor ferrets after inoculation of A/California/7/2009 (A) or A/ruddy turnstone/Delaware/300/2009 (B). Body weights from inoculated animals were collected every day (groups A, C, D, E, G, H, and K) or every other day (groups B, F, I, and J) postinoculation through the days indicated. Body weight percentages were set at 100% on the day of inoculation for each animal; lines represent individual ferrets. Ferrets reaching endpoint criteria after inoculation of A/ruddy turnstone/Delaware/300/2009 (group D and F) were humanely euthanized. Download FIG S2, DOCX file, 0.3 MB. Copyright © 2022 Belser et al. 2022 Belser et al. https://creativecommons.org/licenses/by/4.0/ This content is distributed under the terms of the Creative Commons Attribution 4.0 International license.

### Confidence in virus transmission results generated from multiple laboratories.

**FIG 3:**
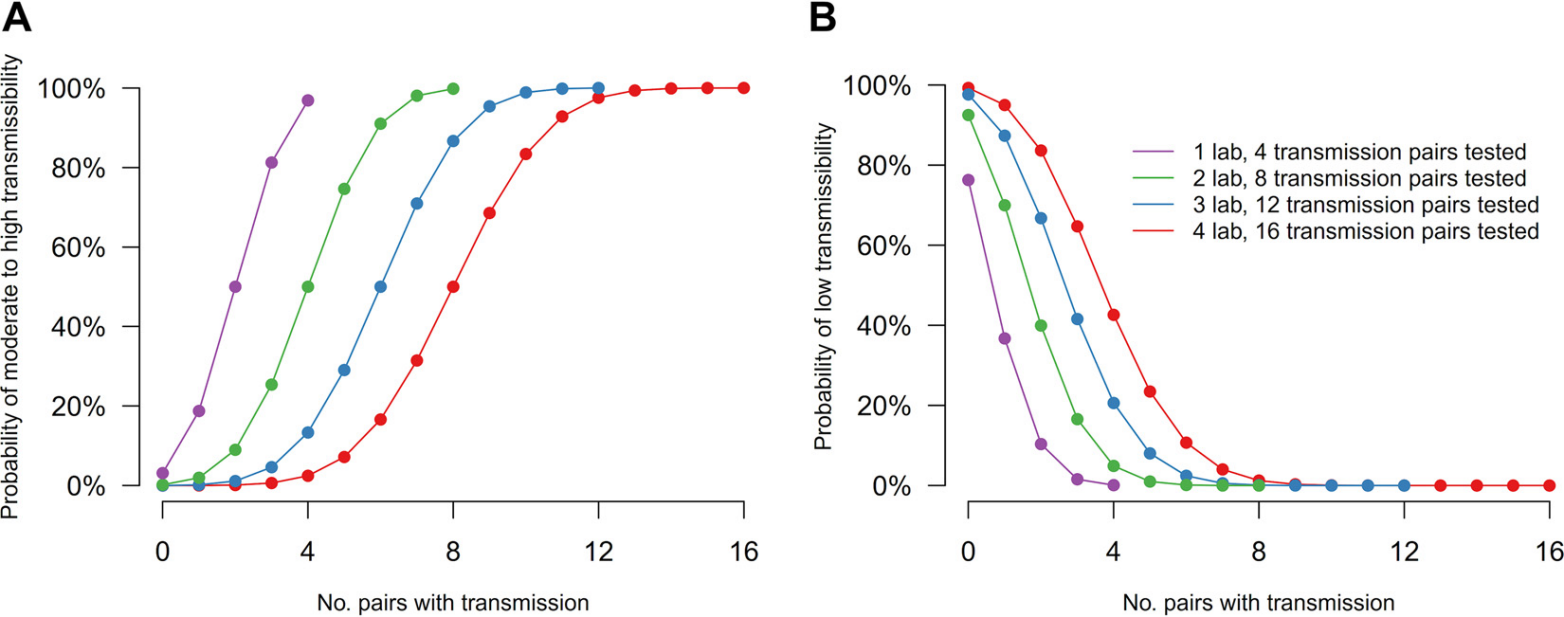
Confidence in conclusions derived from pooled samples from multiple laboratories. (A) Probability to accurately conclude the assessed virus to possess moderate to high transmissibility (*P* of ≥50% among all transmission events). (B) Probability to accurately conclude the assessed virus to possess low transmissibility (*P* of ≤25% among all transmission events). Each laboratory was assumed to provide results from 4 donor-contact pairs at a 1:1 ratio; transmission in each pair is an independent event. Transmission events in contact ferrets (*x* axis) are defined as detection of infectious virus and seroconversion to the exposed virus.

Collectively, the results from this exercise demonstrate a capacity for groups possessing differences in facility designs and experimental protocols to report various levels of relative transmissibility and pathogenicity following inoculation of ferrets with the same virus. To illustrate how confidence in risk assessments of virus transmissibility can increase as results from multiple groups are combined, we evaluated the hypothetical risk of a virus possessing moderate to high transmissibility (defined as *P* of ≥50% transmission events per total ferret pairs) or low transmissibility (defined as *P* of ≤25% transmission events per total ferret pairs). In these analyses, concordant results are defined as multiple groups identifying a virus exhibiting the same transmission capacity, and discordant results are defined as multiple groups identifying a virus with different transmission capacities, as defined above. By assuming concordant results across laboratories, which permits pooling of all transmission outcomes, as few as three groups (12 pairs of ferrets) will yield a probability of over 80% to conclude moderate to high transmissibility when transmission is observed in more than 75% of all experiments and a probability of over 85% to conclude low transmissibility when at most one transmission event is observed over all experiments (Fig. 3). Considering our cross-laboratory exercise from the 11 laboratories and under the same assumption that transmissibility was completely unknown before the experiment, the confidence in concluding moderate to high transmissibility for Cal/09 would be >99.9% (based on 35/44 transmission events), and that in concluding low transmissibility for ruddy turnstone/09 would be 94.8% (based on 6/43 transmission events).

Alternatively, a voting system can be considered by first drawing a conclusion on transmissibility in each laboratory, with an overall conclusion drawn based on these votes from multiple labs. When testing for moderate to high transmissibility, and assuming 4 independent transmission pairs per laboratory, 3 laboratories are needed to conclude moderate to high transmissibility with confidence of >90% if concordant results are obtained (Table 3). In agreement with probabilities shown in Fig. 3, a greater number of laboratories contributing results is needed to demonstrate statistically significant results when testing for low transmissibility. To conclude low transmissibility with >90% confidence, this would necessitate 5 contributing laboratories if concordant results are obtained (Table 3). In this scenario, a greater number of contributing laboratories (or a greater number of donor-contact pairs per laboratory) would be required if the true transmission probability was higher for confirming low transmissibility, or when the true transmission probability was lower for confirming moderate to high transmissibility.

**TABLE 3:** Confidence in conclusions derived from multiple laboratories considering a voting system

No. of labs^*a*^	No. of labs with concordant results^*b*^	Probability (%) of:	
		Moderate to high transmissibility	Low transmissibility
1	1	76	56
2	2	87	72
	1	31	18
3	3	92	81
	2	48	32
4	4	95	87
	3	62	45
	2	23	12
5	5	97	95
	4	72	56
	3	35	21
6	6	98	94
	5	80	65
	4	47	30

a Number of laboratories providing votes on the transmissibility of the tested virus is shown. Each laboratory will vote if the tested virus possesses moderate to high transmissibility (*P* ≥ 50%, i.e., ≥2 infected out of 4 ferrets) or low transmissibility (*P* ≤ 25%, i.e., 0 or 1 infected out of 4 ferrets) based on the experimental result.

b Concordant result that supports either moderate to high or low transmissibility across participating laboratories.

Despite generally consistent results between all groups in this exercise, discordant results are possible (Table 1), highlighting the need to better understand how to responsibly interpret and account for these findings. Therefore, we also considered the scenario when discordant results between laboratories are recorded. To demonstrate moderate to high transmissibility, we found that 6 laboratories with 1 discordant result could still provide 80% confidence in the conclusion, while any discordant result significantly reduced confidence for concluding low transmissibility (Table 3). Considering our cross-laboratory exercise in which experiments from all 11 laboratories were concordant for Cal/09, the confidence in concluding moderate to high transmissibility for Cal/09 would be 99.8% for the voting system. Experiments for ruddy turnstone/09 were concordant from 10 out of 11 laboratories, and the confidence in concluding low transmissibility for would be 90.1%.

In both scenarios, if the results from different laboratories were more heterogeneous, the uncertainty around the conclusion from each lab increases and the overall confidence would decrease. This exercise is an illustration of the possible scenarios and confidence in drawing conclusions on transmissibility but would be affected by how moderate to high or low transmissibility was defined.

## DISCUSSION

The importance of the ferret model for influenza virus risk assessment studies cannot be overstated (12, 40). Recent advances in molecular biology, aerobiology, genomics, and other areas highlight the ways the ferret model in general and studies evaluating virus transmissibility by the airborne route specifically continue to contribute toward our understanding of influenza viruses and the threat they pose to human health (41–43). However, as this model becomes more commonly employed in laboratories worldwide, there is a pressing need to capture the level of variability and heterogeneity intrinsic to this research. Cross-laboratory exercises have been employed in the past to evaluate the reproducibility of assays employed for influenza virus public health efforts (3–6, 44), but no such exercise has been performed to date evaluating influenza virus transmissibility in the ferret. In this exercise, 11 laboratories independently evaluated the ferret-to-ferret transmissibility of Cal/09 and ruddy turnstone/09 viruses that possess distinct transmission potential in humans. With only a few experimental parameters (common virus stock, standardized inoculation dose, route, volume, and the 1:1 donor/contact ratio) being controlled across the participating laboratories, we observed homogenous transmission outcomes (that is, outcomes did not differ statistically) across laboratories. Our results demonstrate the robustness of the ferret model in influenza risk assessment studies.

Risk assessment rubrics have thoroughly evaluated a wide scope of influenza A viruses, from viruses associated with poultry outbreaks in the absence of confirmed human infections to viruses such as A(H5N1) and A(H7N9) influenza viruses, which have caused substantial human disease and death (2, 45). As such, there is a need to evaluate the heterogeneity of ferret transmission models employing viruses possessing a similar scope of transmissibility phenotypes. While the variability in transmission results for either the Cal/09 or ruddy turnstone/09 virus tested in this study were not statistically significant, the range of results obtained, especially with the ruddy turnstone/09 virus, nonetheless illustrates a level of variability that can be present in transmission readouts of viruses exhibiting both low to high transmission efficiency (Table 1). This variability was present despite a high degree of standardization of virus stock, inoculation procedures, and uniformity of donor/contact ratio. It should be noted that the two common virus stocks employed in this study were propagated in different substrates at different institutions. While risk assessment activities encompass viruses propagated in both eggs and cells, justifying the inclusion of both substrates in this study, future studies evaluating the specific contribution of stock passage history to heterogeneity in results generated *in vivo* would be of benefit.

As shown in Text S1 and Tables S2 to S4, this exercise captured the extensive heterogeneity in laboratory protocols and setups present between different groups. Documented variation was present in every parameter examined, inclusive of ferrets, cage setups, titration methods, and environmental conditions, among other features. It is impossible to standardize all contributing variables to these experiments, as institutional, animal welfare, and governmental guidelines and requirements vary worldwide, as do cost implications. That said, this exercise supports the capacity to harmonize results generated between disparate groups when a small number of procedural parameters are fixed. Despite of a great level of variation recorded across laboratories, relative or absolute humidity was not associated with aerosol transmission in a linear or U-shape relationship (46) (Table 2). Caging and airflow considerations were especially variable (Table S3). While directional airflow (odds ratio [OR] = 4) did not reach statistical significance (Table 2), it is nonetheless of note that 3/4 laboratories for which ruddy turnstone/09 virus transmission was detected possessed directional airflow, versus 3/7 of the laboratories for which transmission with this virus was not detected; directional airflow from inoculated to contact animals was a feature in 6/11 laboratories in this exercise. Transmission percentages between the two viruses were highly correlated between laboratories (Spearman correlation = 0.86; *P* < 0.001). Furthermore, there is also significant variation in the age, gender, and suppliers of the ferrets used in this study (Table S2). While all groups conducted hemagglutination inhibition (HI) testing to confirm seronegativity to the H1N1 viruses tested in this study prior to inoculation, we cannot exclude the possibility that low levels of preexisting heterosubtypic immunity may have nonetheless been present that could not be captured by the HI testing employed. Collectively, our results suggest that the airborne transmission phenotype of an influenza virus is multifactorial and that a confluence of parameters may create a more permissive environment for virus transmission to occur.

10.1128/mbio.01174-22.1 TEXT S1 Ethics statement and group specific experimental settings that are not standardized in this study. Download Text S1, DOCX file, 0.02 MB. Copyright © 2022 Belser et al. 2022 Belser et al. https://creativecommons.org/licenses/by/4.0/ This content is distributed under the terms of the Creative Commons Attribution 4.0 International license.

To improve interpretation of results from this standardization exercise, we concurrently investigated the hypothetical confidence in concluding low transmissibility (*P* of ≤25% transmission events per total ferret pairs) or moderate to high transmissibility (*P* of ≥50% transmission events per total ferret pairs) from multiple contributing laboratories. These analyses assumed both a uniform prior distribution for the transmission probability for a novel pathogen and independent transmission outcomes from the laboratories. We considered two scenarios: one scenario where strong homogeneity across laboratories could be assumed so the observations were pooled from multiple laboratories and another scenario where each laboratory drew their own conclusion on transmissibility such that an overall conclusion was drawn as a voting system. As influenza viruses of notable public health importance are frequently assessed across multiple independent laboratories, these analyses provide a framework to rigorously interpret independently generated findings, especially when discordant results between laboratories are reported. This is most critical in the event of a novel virus believed to possess moderate to high transmissibility; our analyses support the notion that the phenomenon of 3 independent laboratories with concordant results supporting an enhanced transmissibility phenotype yields a 92% probability of this finding (Table 3), with additional independent groups or a greater number of total ferret donor-contact pairs necessary when discordant results are present.

Collectively, the findings of this exercise support the potential benefit of increased uniformity, or standardization, of some parameters when conducting risk assessment-specific activities on the same viruses. Specifically, the donor/contact ratio represents such a parameter. For a virus with moderate to high transmissibility, such as Cal/09 virus, modulation of this ratio (e.g., conducting experiments with a 2:1 donor/contact ratio, as is the case when transmission evaluations in a direct-contact setting and via respiratory droplets employ a common donor) would not substantially alter conclusions drawn. However, for a virus with reduced transmissibility at a 1:1 ratio, such as the ruddy turnstone/09 virus evaluated in this work, it is likely that an increased donor/contact ratio (e.g., 2:1) may enhance transmissibility by increasing virus-laden aerosols exhaled from infected ferrets. Previous studies on ruddy turnstone/09 virus demonstrated airborne transmission potential when employing a donor/direct contact/aerosol contact ratio of 1:1:1; efficient transmission by direct contact will subsequently affect the quantity and kinetics of virus-laden aerosols that mediate transmission by air (36, 37). There is a need to better understand how modulation of this ratio contributes to assessments of virus transmissibility. However, this does underscore the potential complications posed by harmonizing data generated for risk assessment purposes for which the donor/contact ratio diverges. With increased heterogeneity in results between labs, uncertainty around the conclusions increases, and there is a corresponding decrease in confidence in the results (Fig. 3 and Table 3), showing the utility in increasing homogeneity across findings from different labs in order to reduce the total number of labs required to yield statistically meaningful results in this sort of analysis.

The emergence of severe acute respiratory syndrome coronavirus 2 (SARS-CoV-2) further corroborates the pandemic potential of viruses of zoonotic origin. Early identification and risk assessments of novel viruses are essential for preventing the next pandemic. Continued optimization and refinement of risk assessment protocols will facilitate data interpretation in response to emerging pandemic threats. Collectively, a greater appreciation of this heterogeneity and understanding of the scope of variability present in risk assessment settings will permit more robust conclusions to be drawn from these efforts in the future.

## MATERIALS AND METHODS

### Viruses.

The A(H1N1)pdm09 virus A/California/07/2009 (Cal/09) was propagated in MDCK cells (passage C3) at the U.S. CDC as described previously (19). The low-pathogenicity avian influenza A(H1N1) virus A/ruddy turnstone/Delaware/300/2009 (ruddy turnstone/09) was propagated in eggs (passage E3) by St. Jude Children’s Research Hospital as described previously (37). Stocks were fully sequenced and tested for exclusivity to rule out the presence of other influenza virus subtypes prior to distribution.

### Animal and experimental variability.

Groups obtained ferrets from multiple vendors and independent breeders from North America, Europe, and Asia, and animals varied in age, gender, health status, and other parameters (Table S2). There were substantial differences between laboratories in the specific caging employed for transmission experiments, distance between cages, airflow directionality between cages, and air changes per hour (Table S3). Anesthesia protocols, sample collection methods, and decontamination procedures to prevent cross-contamination between contact and donor animals varied between groups and are reported in Text S1. All experiments were performed under country-specific legal guidelines and approved institutional-specific animal protocols as specified in Text S1.

### Standardized procedures.

All laboratories received common stock viruses prepared by the CDC and St. Jude Children’s Research Hospital with the shipping temperature recorded. Stock viruses were diluted to 10^6^ PFU in 500 μL of phosphate-buffered saline (PBS) based on predetermined viral titers, and donor ferrets were inoculated intranasally under in-house protocols for anesthesia (Text S1). On day 1 postinoculation, one respiratory droplet contact ferret was introduced and exposed to each donor by housing in an adjacent cage, employing a strict 1:1 donor/contact ratio, with 4 transmission pairs tested for each virus. Ferret temperatures, weights, and nasal washes/swabs were collected every 24 to 48 h. Daily room temperature and relative humidity readings were collected employing prevalidated thermohygrometers (Testo Inc.; 608-H1) that give comparable readings (Table S4). Sera were collected at the end of each experiment for determination of seroconversion to homologous virus by hemagglutination inhibition assay using established in-house serology protocols.

### Sample titration and normalization.

Infectious virus titers were determined by plaque assay, 50% tissue culture infectious dose (TCID_50_) assay, or 50% egg infectious dose (EID_50_) assay at each laboratory, with various limits of detection (Table S1). To facilitate subsequent statistical assessments across laboratories, reported titers from each laboratory were normalized to TCID_50_ per milliliter for each virus based on PFU, TCID_50_, and EID_50_ values predetermined by a single laboratory to minimize titration methodology-specific variation.

### Data blinding and analyses.

Data blinding and aggregation and all statistical analyses were performed by an independent statistician. Transmission outcomes were compared across laboratories by each virus, using Fisher’s exact test of homogeneity. Viral loads between viruses were compared by testing difference in area under the curve (AUC) using *t* test. Factors associated with transmissibility and morbidity were assessed by using logistic regression and linear regression models. We also investigated the confidence in concluding low transmissibility (*P* of ≤25%, or ≤1 ferret infected out of 4 ferrets) or moderate to high transmissibility (*P* of ≥50%, or ≥2 ferrets infected out of 4 ferrets) from multiple contributing laboratories. We assumed a uniform prior distribution for the true transmission probability for a novel pathogen and independent transmission outcomes from the laboratories. The assumed transmission probability determined the likelihood of different observations from the laboratories, based on which the conclusion of low or moderate to high transmissibility was made. For each possible observation of transmission events arising from the assumed true transmission probabilities, we calculated the probability of drawing a correct conclusion, i.e., confidence of the conclusion. The overall confidence was computed by integrating the above-described probabilities over the assumed true transmission probabilities according to the uniform distribution. We considered a scenario where strong homogeneity across laboratory can be assumed so observations of the transmission events between ferret pairs were pooled from multiple laboratories and another scenario where each laboratory drew its own conclusion on transmissibility and the overall conclusion was drawn as the voting system. The confidence of drawing conclusion on transmissibility with the number of observed transmission events among the pooled samples or concordant or discordant outcomes from the laboratories for the voting system is presented. All analyses were conducted in R version 4.0.4 (R Development Core Team).
