# Supplementary material for: Robustness of the Ferret Model for Influenza Risk Assessment Studies: a Cross-Laboratory Exercise
Source: mBio. 2022 Jul 11;13(4):e01174-22. doi: 10.1128/mbio.01174-22 (PMC9426434; doi:10.1128/mbio.01174-22)
Supplement: TABLE S1 [file mbio.01174-22-s0002.docx]

**Supplemental Table 1. Summary of titration methodology by each laboratory.**

| **Group** | **Titers determined in each laboratory** | | | | **After normalization*^d^* (TCID_50_/ml)** | |
| --- | --- | --- | --- | --- | --- | --- |
|  | **Units*^a^*** | **LOD/ml*^b^*** | **Cal/09*^c^*** | **Ruddy turnstone/09*^c^*** | **Cal/09** | **Ruddy turnstone/09** |
| A | PFU | 10 | 9.0x10^6^ | 6.6x10^5^ | 1.8x10^7^ | 1.32x10^6^ |
| B | EID_50_ | 5.62 | 2.51x10^6^ | 2.29x10^7^ | 4.46x10^6^ | 4.07x10^7^ |
| C | TCID_50_ | 10 | 1.0x10^5^ | 1.58x10^6^ | 1.0x10^5^ | 1.58x10^6^ |
| D | PFU | 10 | 7.0x10^7^ | 1.65x10^7^ | 1.4x10^8^ | 3.30x10^7^ |
| E | TCID_50_ | 100 | 1.86x10^6^ | 9.33x10^5^ | 1.86x10^6^ | 9.33x10^5^ |
| F | TCID_50_ | 3.16 | 6.31x10^6^ | 1.0x10^7^ | 6.31x10^6^ | 1.0x10^7^ |
| G | TCID_50_ | 10 | 5.0x10^6^ | 2.5x10^6^ | 5.0x10^6^ | 2.5x10^6^ |
| H | PFU | 10 | 7.0x10^6^ | 2.6x10^6^ | 1.4x10^7^ | 5.2x10^6^ |
| I | TCID_50_ | 61.5 | 4.9x10^7^ | 6.0x10^7^ | 4.9x10^7^ | 6.0x10^7^ |
| J | TCID_50_ | 3.16 | 1.58x10^7^ | 5.62x10^5^ | 1.58x10^7^ | 5.62x10^5^ |
| K | TCID_50_ | 3.16 | 2.82x10^6^ | 1.58x10^6^ | 2.82x10^6^ | 1.58x10^6^ |

*^a^*PFU, plaque forming units. EID_50_, 50% egg infectious dose. TCID_50_, 50% tissue culture infectious dose (employing MDCK cells). *^b^*LOD/ml, limit of detection for titration of ferret specimens for the units specified. *^c^*Titration of received virus employing laboratory assay-specific methods. *^d^*For standardization purposes, all infectious titers were normalized to TCID_50_/mL based on pre-determined titers for the Cal/09 virus (5x10^6^ PFU/ml, 5.01x10^6^ EID_50_/ml, and 1.0x10^7^ TCID_50_/ml), and ruddy turnstone/09 virus (2.5x10^6^ PFU/ml, 3.16x10^8^ EID_50_/ml, and 3.16x10^6^ TCID_50_/ml).
