# Supplementary material for: Robustness of the Ferret Model for Influenza Risk Assessment Studies: a Cross-Laboratory Exercise
Source: mBio. 2022 Jul 11;13(4):e01174-22. doi: 10.1128/mbio.01174-22 (PMC9426434; doi:10.1128/mbio.01174-22)

**Supplemental Figure 1. Transmission kinetics of H1N1 viruses.** Nasal washes (all groups except Group F) or throat swabs (Group F) were sampled from donor (left bars) and aerosol contact ferrets (right bars) to determine infectious viral loads following inoculation with A/California/7/2009 (A) or A/ruddy turnstone/DE/300/2009 (B); titers are reported as log_10_ PFU/ml (Groups A, D, H), log_10_ TCID_50_/ml (Groups C, E, F, G, I, J, K), or log_10_ EID_50_/ml (Group B). Limit of detection for each graph is reported in Supplemental Table 1.


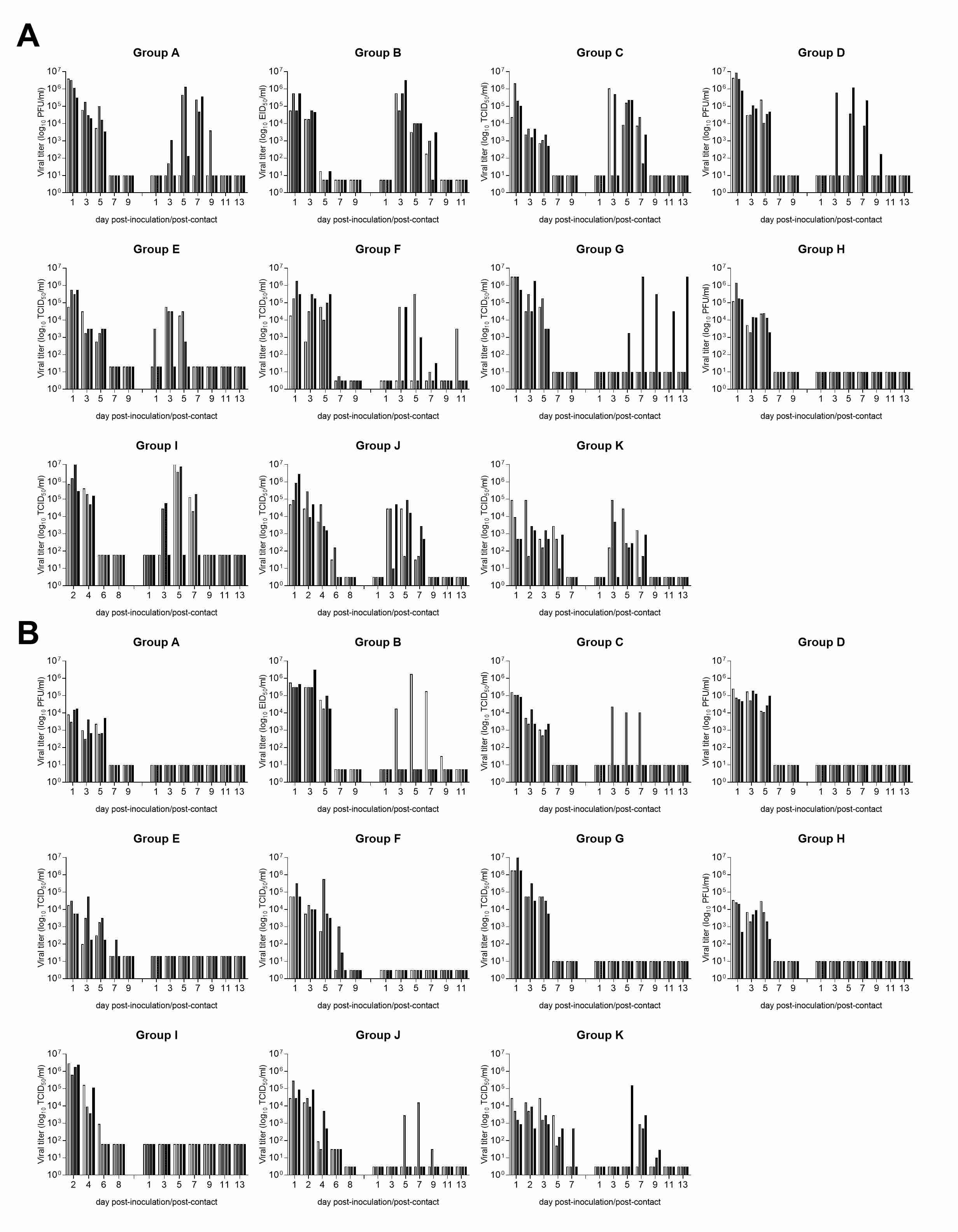

Supplement: FIG S1 [file mbio.01174-22-s0009.docx]
