# Supplementary material for: Robustness of the Ferret Model for Influenza Risk Assessment Studies: a Cross-Laboratory Exercise
Source: mBio. 2022 Jul 11;13(4):e01174-22. doi: 10.1128/mbio.01174-22 (PMC9426434; doi:10.1128/mbio.01174-22)
Supplement: TABLE S2 [file mbio.01174-22-s0003.docx]

**Supplemental Table 2. Ferret source and health status prior to study.**

| **Group** | **Commercial vendor** | **Spayed/neutered/descented** | **Gender** | **Vaccination status** | **Additional treatments** |
| --- | --- | --- | --- | --- | --- |
| A | Triple F Farms, USA | Yes | M | rabies, distemper | none |
| B | Independent breeders | Yes | F | distemper, parvovirus | none |
| C | Triple F Farms, USA | Yes | F | rabies, distemper | meloxicam, penicillin, ivermectin, ponazril |
| D | Triple F Farms, USA | Yes | F | rabies, distemper | meloxicam, penicillin, ivermectin, ponazril, |
| E | Independent breeders | Yes (M only) | M/F | distemper | hormone blocker (F only) |
| F | Triple F Farms, USA | Yes | F | rabies | none |
| G | Triple F Farms, USA | Yes | M | rabies, distemper | none |
| H | Highgate Farms, UK | No | F | none | delvosteron (F only) |
| I | Independent breeders | Yes | M | rabies, distemper, parvovirus | none |
| J | Triple F Farms, USA | Yes | M/F | rabies, distemper | none |
| K | Triple F Farms, USA | Yes | M | rabies, distemper | none |
