# Supplementary material for: Robustness of the Ferret Model for Influenza Risk Assessment Studies: a Cross-Laboratory Exercise
Source: mBio. 2022 Jul 11;13(4):e01174-22. doi: 10.1128/mbio.01174-22 (PMC9426434; doi:10.1128/mbio.01174-22)
Supplement: TABLE S3 [file mbio.01174-22-s0004.docx]

**Supplemental Table 3. Caging and physical environment.**

| **Group** | **cage dimensions LxWxH (cm)*^a^*** | **cage material** | **cage interface distance** | **transmission interface** | **airflow to contacts*^b^*** | **ACH*^c^*** |
| --- | --- | --- | --- | --- | --- | --- |
| A | 56 x 42 x 42 | stainless steel | 0.3-1 cm | two perforated side-walls with openings <5 mm | no | 150-180 |
| B | 25 x 33 x 40 | stainless steel | 4 cm | double-layered net divider | yes | 20-50 |
| C | 36 x 36 x 41 | stainless steel | 5 cm | two perforated side-walls with openings 13 mm | no | 12-15 |
| D | 28 x 38 x 30 | stainless steel | 5 cm | stainless steel mesh | no | 45 |
| E | 28.3 x 78.7 x 41 | stainless steel | 2 cm | double-layered stainless steel perforated divider with openings 5 mm | yes | 36-44 |
| F | 50 x 30 x 30 | perspex | 10 cm | perforated stainless steel grid with openings 10 mm | yes | 30-40 |
| G | 72 x 61 x 41 | stainless steel | 0.5-1 cm | two perforated side-walls with openings 5 mm | no | 15 |
| H | 78 x 50 x 50 | perspex/stainless steel | 0.25 cm | perforated stainless steel grid with openings 5 mm | no | 15 |
| I | 61 x 61 x 48 | stainless steel | 4.5-5 cm | perforated stainless steel grid with openings 5 mm | yes | 39-41 |
| J | 68.6 x 28 x 40.6 | stainless steel | 2.5 cm | two perforated side-walls with openings 5 mm | yes | 25 |
| K | 28 x 79 x 41 | stainless steel | 2.5 cm | two perforated panels with staggered openings 5mm | yes | 25-35 |

*^a^*Housing environment of individually-housed ferrets. *^b^*Yes, directional airflow from inoculated to contact cages; No, airflow is ambient or otherwise ot directional from inoculated to contact cages. *^c^*ACH, air changes per hour within primary containment area.
