# Supplementary material for: Robustness of the Ferret Model for Influenza Risk Assessment Studies: a Cross-Laboratory Exercise
Source: mBio. 2022 Jul 11;13(4):e01174-22. doi: 10.1128/mbio.01174-22 (PMC9426434; doi:10.1128/mbio.01174-22)
Supplement: TABLE S4 [file mbio.01174-22-s0005.docx]

**Supplemental Table 4. Environmental summaries for transmission experiments.**

| **Group** | **Virus** | **temperature (°C)** | | **relative humidity (% RH)^a^** | |
| --- | --- | --- | --- | --- | --- |
|  |  | **mean** | **range** | **mean** | **range** |
| A | Cal/09 | 21.1 | 21.0-21.2 | 54.1 | 53.5-55.0 |
|  | Ruddy turnstone/09 | 21.0 | 20.2-21.2 | 54.6 | 51.9-56.7 |
| B | Cal/09 | 21.4 | 21.2-22.8 | 32.9 | 30.2-38.5 |
|  | Ruddy turnstone/09 | 21.4 | 21.2-22.8 | 32.9 | 30.2-38.5 |
| C | Cal/09 | 22.2 | 20.7-24.1 | 77.0 | 61.0-86.5 |
|  | Ruddy turnstone/09 | 22.0 | 21.0-23.4 | 67.5 | 39.9-99.6 |
| D | Cal/09 | 23.2 | 23.0-24.1 | 45.0 | 42.7-46.6 |
|  | Ruddy turnstone/09 | 23.2 | 23.0-24.1 | 45.0 | 42.7-46.6 |
| E | Cal/09 | 21.4 | 21.0-21.9 | 52.9 | 39.0-59.0 |
|  | Ruddy turnstone/09 | 21.6 | 20.9-22.0 | 51.9 | 43.8-64.5 |
| F | Cal/09 | 22.0 | 21.1-22.3 | 44.7 | 42.0-49.3 |
|  | Ruddy turnstone/09 | 21.9 | 21.4-22.2 | 45.2 | 41.2-54.7 |
| G | Cal/09 | 22.3 | 21.7-23.5 | 47.5 | 41.2-59.4 |
|  | Ruddy turnstone/09 | 22.3 | 21.7-23.5 | 47.5 | 41.2-59.4 |
| H | Cal/09 | 22.2 | 20.7-24.7 | 63.8 | 58.9-73.1 |
|  | Ruddy turnstone/09 | 21.1 | 20.6-21.8 | 69.8 | 64.8-76.7 |
| I | Cal/09 | 20.5 | 20.2-21.1 | 55.3 | 37.7-67.0 |
|  | Ruddy turnstone/09 | 20.5 | 20.2-21.1 | 55.3 | 37.7-67.0 |
| J | Cal/09 | 22.9 | 22.1-23.6 | 40.8 | 36.0-49.4 |
|  | Ruddy turnstone/09 | 21.7 | 20.8-23.2 | 32.7 | 32.0-33.0 |
| K | Cal/09 | 21.9 | 21.2-22.3 | 47.8 | 45.1-54.9 |
|  | Ruddy turnstone/09 | 21.8 | 21.3-22.2 | 48.3 | 43.8-59.1 |

^a^ Pre-validated hygrometers with comparable readings were employed to measure temperature and relative humidity in each facility; hygrometers were pre-calibrated at a central location prior to distribution to all participating groups. All groups employ a 12-hour light/dark cycle with the exception of Group E, which employs a 9-hour light/dark cycle.
