# Supplementary material for: Robustness of the Ferret Model for Influenza Risk Assessment Studies: a Cross-Laboratory Exercise
Source: mBio. 2022 Jul 11;13(4):e01174-22. doi: 10.1128/mbio.01174-22 (PMC9426434; doi:10.1128/mbio.01174-22)
Supplement: TABLE S5 [file mbio.01174-22-s0006.docx]

**Supplemental Table 5. Clinical signs of donor ferrets following inoculation with Cal/09 virus.**

| Group | Gender | Age (months) | Body weight changes*^a^* | | | Temperature*^a^* | | | Respiratory signs*^e^* | Lethality*^f^* | RII*^g^* |
| --- | --- | --- | --- | --- | --- | --- | --- | --- | --- | --- | --- |
|  |  |  | Baseline (g)*^b^* | Mean max loss (%)*^c^* | Range (day) *^d^* | Baseline (°C) *^b^* | Mean max rise (°C) *^c^* | Range (day) *^d^* |  |  |  |
| A | M | 8 | 1465 | 8.5 | 6-9 | 38.2 | 1.8 | 1-6 | 2/4 | 0/4 | 1.00 |
| B | F | 4-6 | 961 | 5.0 | 6 | 38.3 | 0.7 | 2 | 4/4 | 0/4 | 1.27 |
| C | F | 5 | 688 | 16.9 | 3-12 | 39.7 | 0.6 (3/4) | 2-11 | 4/4 | 0/4 | 1.66 |
| D | F | 5 | 699 | 15.6 | 6-11 | 37.2 | 2.1 | 5-14 | 0/4 | 0/4 | 1.00 |
| E | M/F | 4 | 862 | 4.9 | 4-11 | 37.8 | 1.9 | 1-4 | 4/4 | 0/4 | 1.04 |
| F | F | 6-12 | 829 | 9.3 | 3-7 | 38.4 | 1.0 | 1-5 | 2/4 | 0/4 | 1.39 |
| G | M | 3-5 | 1013 | 8.6 | 1-6 | 38.4 | 1.0 | 1-4 | 3/4 | 0/4 | 1.21 |
| H*^h^* | F | 4-5 | 905 | 3.9 (3/4) | 2-6 | 38.2 | 0.7 | 2-10 | 3/4 | 0/4 | 1.05 |
| I | M | 4-6 | 1258 | 1.0 (1/4) | 2 | 39.1 | 0.7 | 2 | 4/4 | 0/4 | 1.07 |
| J | M/F | 6 | 1144 | 11.2 | 5-6 | 39.0 | 0.8 | 1-10 | 0/4 | 0/4 | 1.30 |
| K | M | 6-8 | 1399 | 11.5 | 6-11 | 38.1 | 1.6 | 2-5 | 1/4 | 0/4 | 1.04 |

*^a^*Ferret data are inclusive of n=4 unless otherwise specified. Data are reflective of measurements collected every 24 hrs (Groups A, C, D, E, G, H, K) or 48 hrs (Groups B, F, I, J). *^b^*Mean pre-inoculation body weight (in grams) or temperature (in °C). *^c^*Percentage mean maximum weight loss or mean maximum rise in temperature (in °C) (compared to baseline on day 0), detected between days 1-14 post-inoculation. Data is inclusive of all ferrets for which weight loss/temperature increases were detected during the observation period; the number of ferrets included in this mean is specified when this is not n=4. *^d^*Day range of maximum weight loss values reported among ferrets included in the reported mean. *^e^*Number of ferrets for which respiratory signs (sneezing, coughing, heavy breathing, open mouth breathing, or nasal discharge) were observed between days 1-14 post-inoculation at least once. *^f^*Number of ferrets that reached humane euthanasia endpoints (day of death specified in parentheses). *^g^*RII, relative inactivity index. *^h^*Reported values for this group span days 0-10 post-inoculation.
