# Supplementary material for: Robustness of the Ferret Model for Influenza Risk Assessment Studies: a Cross-Laboratory Exercise
Source: mBio. 2022 Jul 11;13(4):e01174-22. doi: 10.1128/mbio.01174-22 (PMC9426434; doi:10.1128/mbio.01174-22)
Supplement: TABLE S6 [file mbio.01174-22-s0007.docx]

**Supplemental Table 6. Clinical signs of donor ferrets following inoculation with ruddy turnstone/09 virus.**

| Group | Gender | Age (months) | Body weight*^a^* | | | Temperature*^a^* | | | Respiratory signs*^e^* | Lethality*^f^* | RII*^g^* |
| --- | --- | --- | --- | --- | --- | --- | --- | --- | --- | --- | --- |
|  |  |  | Baseline (g)*^b^* | Mean max loss (%)*^c^* | Range (day) *^d^* | Baseline (°C) *^b^* | Mean max rise (°C) *^c^* | Range (day) *^d^* |  |  |  |
| A | M | 9 | 1415 | 10.7 | 2-11 | 38.5 | 2.0 | 1-4 | 1/4 | 0/4 | 1.09 |
| B | F | 4-6 | 1005 | 1.2 | 4-8 | 38.4 | 0.5 | 2 | 3/4 | 0/4 | 1.13 |
| C | F | 5 | 628 | 12.9 | 1-8 | 39.2 | 0.6 (3/4) | 1-2 | 3/4 | 0/4 | 1.59 |
| D | F | 5 | 678 | 17.8 | 6-12 | 37.3 | 3.0 | 2 | 0/4 | 2/4 (12) | 1.31 |
| E | F | 4 | 809 | 11.4 | 2-9 | 38.3 | 1.5 | 1-2 | 4/4 | 0/4 | 1.13 |
| F | F | 6-12 | 708 | 15.0 | 3-11 | 38.9 | 1.4 (3/4) | 1-5 | 2/4 | 1/4 (7) | 1.39 |
| G | M | 3-5 | 1050 | 16.7 | 2-5 | 37.7 | 1.9 | 2-5 | 2/4 | 0/4 | 1.27 |
| H*^h^* | F | 4-5 | 838 | 7.4 | 5-8 | 38.4 | 0.8 (3/4) | 2-10 | 4/4 | 0/4 | 1.73 |
| I | M | 4-6 | 1220 | 2.2 (2/4) | 2 | 39.2 | 1.2 | 2-4 | 4/4 | 0/4 | 1.23 |
| J | M/F | 6 | 1111 | 12.8 | 2-10 | 38.5 | 2.3 | 1-4 | 0/4 | 0/4 | 1.09 |
| K | M | 6-8 | 1274 | 19.6 | 7-9 | 39.0 | 1.6 | 1-5 | 1/4 | 0/4 | 1.23 |

*^a^*Ferret data are inclusive of n=4 unless otherwise specified. Data are reflective of measurements collected every 24 hrs (Groups A, C, D, E, G, H, K) or 48 hrs (Groups B, F, I, J). *^b^*Mean pre-inoculation body weight (in grams) or temperature (in °C). *^c^*Percentage mean maximum weight loss or mean maximum rise in temperature (in °C) (compared to baseline on day 0), detected between days 1-14 post-inoculation. Data is inclusive of all ferrets for which weight loss/temperature increases were detected during the observation period; the number of ferrets included in this mean is specified when this is not n=4. *^d^*Day range of maximum values reported among ferrets included in the reported mean. *^e^*Number of ferrets for which respiratory signs (sneezing, coughing, heavy breathing, open mouth breathing, or nasal discharge) were observed between days 1-14 post-inoculation at least once. *^f^*Number of ferrets that reached humane euthanasia endpoints (day of death specified in parentheses). *^g^*RII, relative inactivity index. *^h^*Reported values for this group span days 0-10 post-inoculation.
