# Supplementary material for: Robustness of the Ferret Model for Influenza Risk Assessment Studies: a Cross-Laboratory Exercise
Source: mBio. 2022 Jul 11;13(4):e01174-22. doi: 10.1128/mbio.01174-22 (PMC9426434; doi:10.1128/mbio.01174-22)
Supplement: TABLE S7 [file mbio.01174-22-s0008.docx]

**Supplemental Table 7. Parameters associated with % weight changes of Cal/09 and ruddy turnstone/09 inoculated donors by univariable linear regression.**

| **Parameters^a^** | **Cal/09** | | **Ruddy turnstone/09** | |
| --- | --- | --- | --- | --- |
|  | **Difference (95% CI)** | **p-value** | **Difference (95% CI)** | **p-value** |
| Donor viral load (log_10_ AUC)^b^ | 1.6 (-2.5, 5.7) | 0.468 | -0.4 (-5.1, 4.4) | 0.878 |
| Donor peak titer (log_10_TCID_50_/ml) | 0.3 (-1.4, 2.0) | 0.739 | -0.2 (-2.1, 1.7) | 0.826 |
| Donor time to peak titer (dpi) | -2.2 (-11.7, 7.3) | 0.661 | -0.9 (-10.5, 8.6) | 0.852 |
| Gender  (reference: male)^c^ | -2.7 (-9.8, 4.3) | 0.472 | 1.4 (-7.9, 10.8) | 0.771 |
| Air change (per 10 ACH) | 0.1 (-0.7, 0.8) | 0.848 | 0.1 (-0.8, 1.0) | 0.816 |
| Directional airflow to contact  (reference: without directional airflow) | 3.6 (-2.1, 9.2) | 0.251 | 2.7 (-4.5, 10.0) | 0.480 |
| Temperature (°C) | -4.4 (-7.3, -1.5) | 0.015 | -6.2 (-9.9, -2.5) | 0.009 |
| Relative humidity (%) | -0.1 (-0.3, 0.2) | 0.618 | 0.0 (-0.3, 0.4) | 0.897 |
| Absolute humidity (g/m^3^) | -0.6 (-2.0, 0.7) | 0.365 | -0.2 (-2.0, 1.5) | 0.794 |
| Distance between cages (cm) | -0.4 (-1.5, 0.7) | 0.514 | -0.1 (-1.5, 1.3) | 0.884 |

^a^All parameters, except “directional airflow to contact” and gender were numeric. ^b^Also insignificant for viral load (AUC) with P-values = 0.283 and 0.734 for Cal/09 and ruddy turnstone/09 respectively. ^c^Excluded two laboratories which used both genders.
