# Supplementary material for: Robustness of the Ferret Model for Influenza Risk Assessment Studies: a Cross-Laboratory Exercise
Source: mBio. 2022 Jul 11;13(4):e01174-22. doi: 10.1128/mbio.01174-22 (PMC9426434; doi:10.1128/mbio.01174-22)
Supplement: FIG S2 [file mbio.01174-22-s0010.docx]

**Supplemental Figure 2. Weight changes of donor ferrets after inoculation of (A) A/California/7/2009 or (B) A/ruddy turnstone/Delaware/300/2009.** Body weights from inoculated animals were collected every day (Groups A, C, D, E, G, H, K) or every-other-day (Groups B, F, I, J) post-inoculation through the days indicated. Body weight percentages were set at 100% on the day of inoculation for each animal; lines represent individual ferrets. Ferrets reaching endpoint criteria after inoculation of A/ruddy turnstone/Delaware/300/2009 (Group D, F) were humanely euthanized.

**
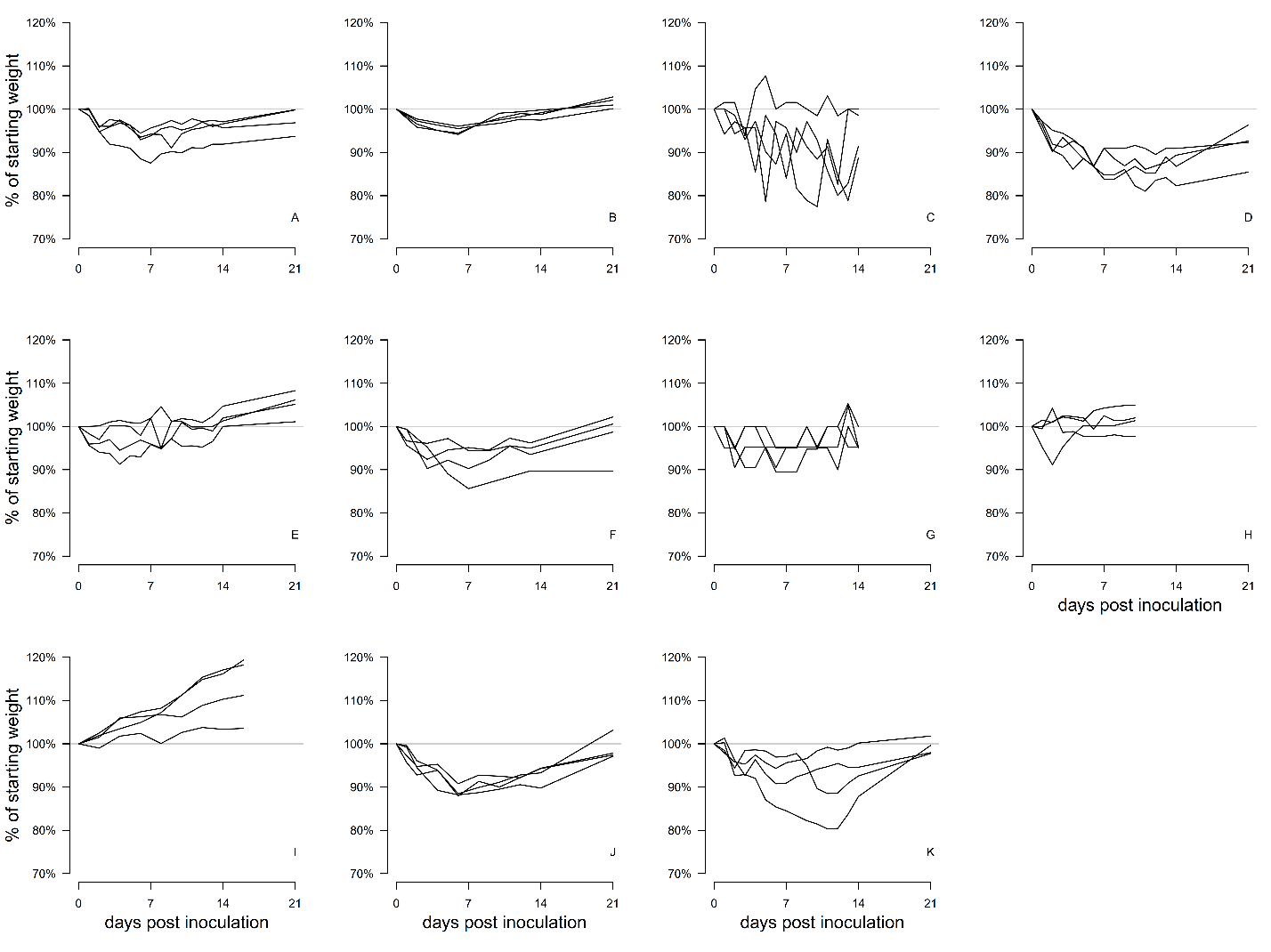
**

**A.**


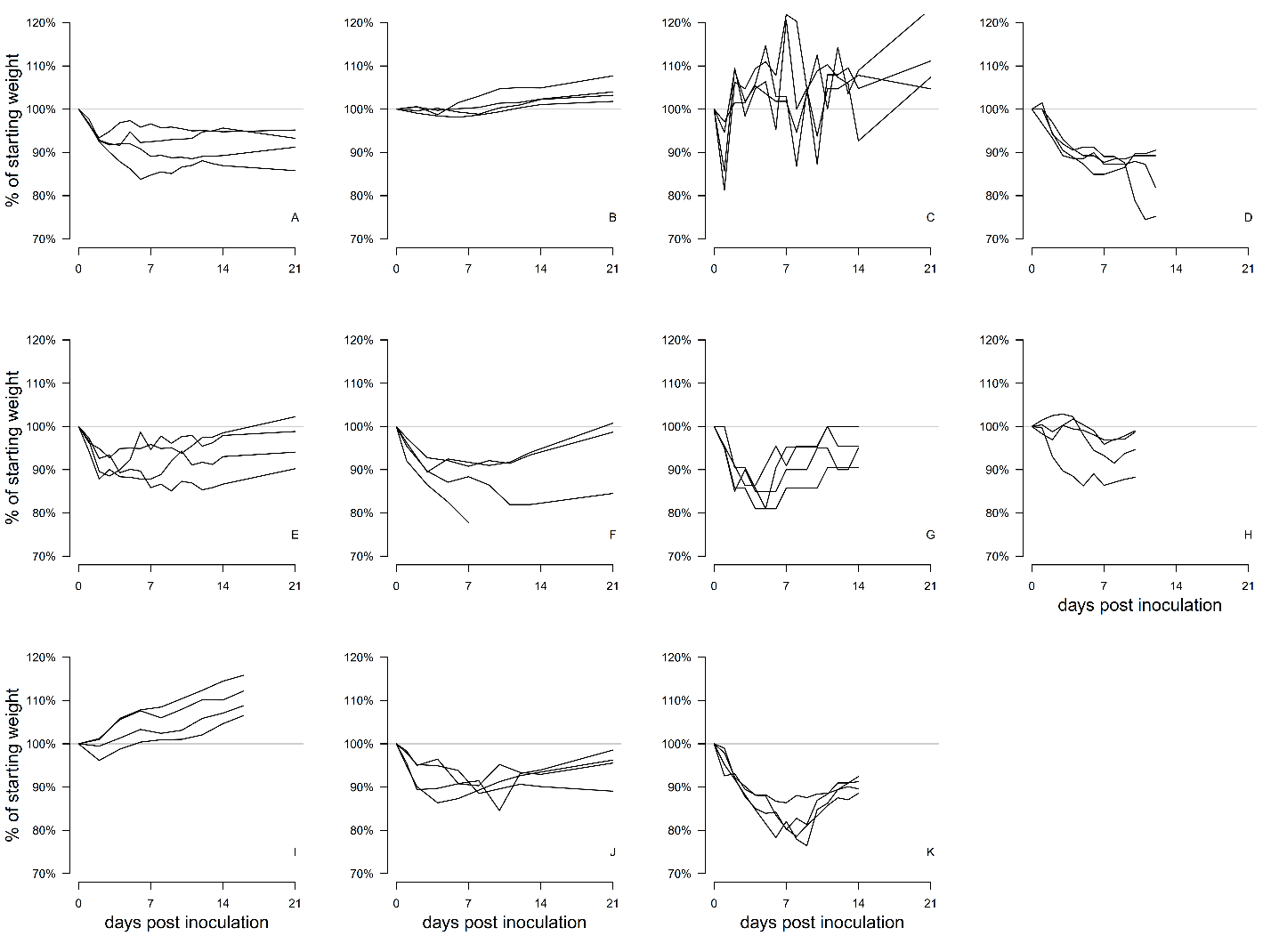
**B**.
